# Urolithin A Ameliorates the TGF Beta-Dependent Impairment of Podocytes Exposed to High Glucose

**DOI:** 10.3390/jpm14090914

**Published:** 2024-08-28

**Authors:** Barbara Lewko, Milena Wodzińska, Agnieszka Daca, Agata Płoska, Katarzyna Obremska, Leszek Kalinowski

**Affiliations:** 1 Department of Pharmaceutical Pathophysiology, Faculty of Pharmacy, Medical University of Gdansk, 80-210 Gdansk, Poland; 2 Independent Researcher, 80-299 Gdansk, Poland; milena.kotewicz@gmail.com; 3 Department of Physiopathology, Faculty of Medicine, Medical University of Gdansk, 80-210 Gdansk, Poland; agnieszka.daca@gumed.edu.pl; 4 Department of Medical Laboratory Diagnostics-Fahrenheit Biobank BBMRI, Faculty of Pharmacy, Medical University of Gdansk, 80-210 Gdansk, Poland; agata.ploska@gumed.edu.pl; 5 Independent Researcher, 10-059 Olsztyn, Poland; obremska.katarzyna204@gmail.com; 6 BioTechMed Center, Department of Mechanics of Materials and Structures, Gdansk University of Technology, 80-223 Gdansk, Poland

**Keywords:** podocytes, podocyte migration, urolithin A, TGF-β1, high glucose, EMT, diabetic nephropathy

## Abstract

Increased activity of transforming growth factor-beta (TGF-β) is a key factor mediating kidney impairment in diabetes. Glomerular podocytes, the crucial component of the renal filter, are a direct target of TGF-β action, resulting in irreversible cell loss and progression of chronic kidney disease (CKD). Urolithin A (UA) is a member of the family of polyphenol metabolites produced by gut microbiota from ellagitannins and ellagic acid-rich foods. The broad spectrum of biological activities of UA makes it a promising candidate for the treatment of podocyte disorders. In this in vitro study, we investigated whether UA influences the changes exerted in podocytes by TGF-β and high glucose. Following a 7-day incubation in normal (NG, 5.5 mM) or high (HG, 25 mM) glucose, the cells were treated with UA and/or TGF-β1 for 24 h. HG and TGF-β1, each independent and in concert reduced expression of nephrin, increased podocyte motility, and up-regulated expression of b3 integrin and fibronectin. These typical-for-epithelial-to-mesenchymal transition (EMT) effects were inhibited by UA in both HG and NG conditions. UA also reduced the typically elevated HG expression of TGF-β receptors and activation of the TGF-β signal transducer Smad2. Our results indicate that in podocytes cultured in conditions mimicking the diabetic milieu, UA inhibits and reverses changes underlying podocytopenia in diabetic kidneys. Hence, UA should be considered as a potential therapeutic agent in podocytopathies.

## 1. Introduction

Diabetic nephropathy (DN) is the leading cause of end-stage kidney disease and accumulating data show that damage of the glomerular filter resulting in proteinuria is one of the major mechanisms of renal impairment [1,2]. It is now widely acknowledged that within the glomerular filtration barrier, podocytes play a pivotal role in controlling the passage of proteins into the urinary space [3,4], and the onset of albuminuria in DN reflects diabetic podocytopathy [5,6].

Podocytes are unique, highly specialized terminally differentiated cells of epithelial origin found in the kidney glomeruli. Diverse biological as well as structural properties of podocytes make them a crucial component of the renal filter [7]. Apart from mechanically supporting the integrity of the glomerular tuft, podocytes are involved in the synthesis and repair of all components of the glomerular filter [7,8]. The cells are anchored to the glomerular basement membrane (GBM) by interdigitating foot processes. The gaps between the neighboring protrusions are bridged by slit diaphragms (SD) that constitute the final barrier for protein loss during filtration through the capillary wall. Podocyte structure, function, and intercellular contact, as well as control of proteinuria, are strictly associated with SD integrity [9]. Despite their critical role in maintaining normal renal function, mature kidney podocytes have a limited ability to regenerate in response to injury, which results in permanent alterations in glomerular structure. This is why podocyte damage, detachment, and loss are considered to be pivotal steps toward progressive chronic kidney disease [10,11].

The transforming growth factor-beta (TGF-β) family of pleiotropic cytokines consists of three (TGF-β1, TGF-β2, and TGF-β3) isoforms, of which TGF-β1 has been established as the predominant isoform expressed in the kidney [12,13]. In physiological conditions, TGF-β1 maintains tissue homeostasis by regulating a broad range of cellular processes and interactions of the cells with the extracellular environment [14,15]. However, excessive TGF-β activity is implicated in the pathogenesis of various diseases by contributing to changes in tissue structure, immunity, redox balance, motility of the cells, and many other features [16,17]. Nearly all kidney diseases are associated with TGF-β1 upregulation, and, in DN, it plays a key role in the development of pathogenic changes in renal tissues [18]. Moreover, many lines of evidence have shown that TGF-β1 is a central mediator of podocyte injury [19,20,21]. In the diabetic kidney, various stimuli such as hyperglycemia, reactive oxygen species (ROS), angiotensin II (Ang II), thrombospondin-1 (TSP-1), and advanced glycation end products (AGEs) induce TGF-β1 synthesis and activate TGF-β1-dependent signaling, which results in diverse injurious changes underlying DN. Also, podocytes, the most vulnerable renal cells, become targets for the overactive TGF-β1 system. This results in a series of functional, morphological, and phenotypic changes, leading to irreversible podocyte impairment and loss [22]. To date, there is no cure for diabetic podocytopathy.

**Figure 1:**
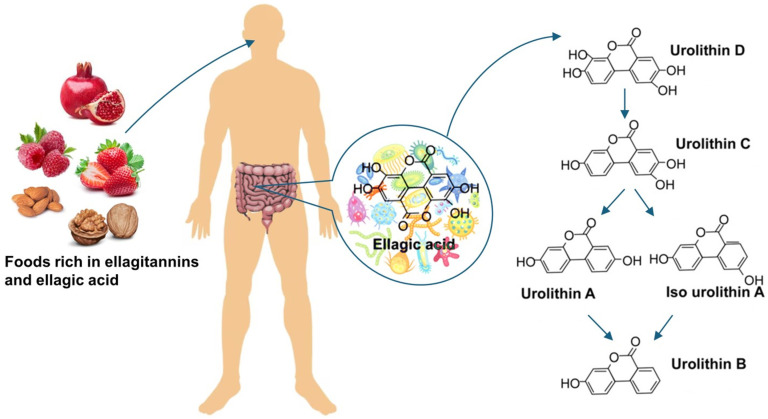
Ellagitannins (ETs) and ellagic acid (EA) are naturally occurring polyphenolic bioactive compounds found in fruits and seeds of various food plants. ETs are hydrolyzed to EA in the upper part of the gastrointestinal tract and further converted by microbiota in the large intestine into urolithins. Depending on individual microbiota composition, various urolithin isoforms are produced, of which urolithin A is the most common form [28]. In contrast to ETs and EA, urolithins are easily absorbed in the gut.

Urolithins, the dibenzo[b,d]pyran-6-one derivatives, are polyphenol metabolites that are produced by the human gut microbiota from ellagitannins and ellagic acid-rich food products such as nuts, pomegranate, and berries (Figure 1). The family consists of several isoforms, of which urolithin A (UA) is the most abundant form in humans [23]. Studies have shown that urolithins, and particularly UA, exhibit various biological activities including antioxidant, anticancer, anti-inflammatory, and antiglycative properties [24,25]. The wide range of these beneficial effects is mediated by diverse urolithin-mediated intracellular mechanisms, such as modulation of apoptosis, signal transduction, cell cycle, gene expression, and others [26]. However, detailed knowledge of the interactions between urolithins and systems regulating cell functions in different organs still remains incomplete. The presence of urolithins in urine indicates their direct contact with renal tissue. Nevertheless, only a few studies on the effects of urolithins in the kidney have been published so far. We have shown recently that UA improved the viability of podocytes exposed to high glucose, and, additionally, the expression and cellular localization of nephrin, the central component of SD, was modulated by this compound [27].

In the present study, we aimed to investigate whether UA can ameliorate injury of podocytes under conditions mimicking diabetes by influencing the activity of TGF-β1.

## 2. Materials and Methods

### 2.1. Podocyte Culture and Treatment

Conditionally immortalized mouse podocytes (SVI clone, Cell Line Services, Eppelheim, Germany) were cultured as described previously [29], with minor modifications. Briefly, the cells were propagated at 33 °C in RPMI 1640 medium (PAN-Biotech, Aidenbach, Germany) containing 11 mM glucose and supplemented with 10% heat-inactivated fetal bovine serum (FBS, EURx, Gdansk, Poland), 100 U/mL penicillin, 100 µg/mL streptomycin (PAN-Biotech, Aidenbach, Germany), and 10 U/mL recombinant mouse interferon-γ (IFN-γ, PeproTech EC, London, UK). Differentiation was induced by shifting the temperature to 37 °C, removing IFN-γ, and changing the medium to DMEM containing 5.5 mM glucose (PAN-Biotech, Aidenbach, Germany) and 5% FBS. After 7–10 days of culture, the cells were divided into two groups. One group remained in DMEM with normal glucose (5.5 mM, NG), while the other group was switched to high glucose (25 mM, HG), and the culture was continued for the next 7 days. Experimental NG or HG media containing 0.5% FBS, 10 mM urolithin A, and 5 ng/mL TGF-β1 were added for the last 24 h. Cells between 18 and 29 passages were used in all experiments. The final concentration of DMSO (dimethyl sulfoxide, solvent for UA) was 0.01% (*v*/*v*) (Merck, Darmstadt, Germany). The effect of the vehiculum was tested in all experiments and no significant changes were observed.

### 2.2. Urolithin A

Urolithin A (UA, 3,8-dihydroxy-6H-dibenzo[b,d]pyran-6-one) was synthesized in the Department of Organic Chemistry of the Medical University of Gdansk, based on literature data [30], and was kindly provided by the Department of Pharmacognosy and Department of Organic Chemistry, Medical University of Gdansk, Poland. UA (228.2 g/mol) was dissolved in sterile dimethyl sulfoxide and 10 mM stock solution was stored at −80 °C.

### 2.3. Podocyte Migration Assay

Differentiated podocytes were cultured in 12-well culture plates. Immediately after adding experimental media containing tested compounds, the cell monolayers were scratched with a 10 mL pipette tip and incubations were held for the next 24 h. Fixed with buffered 2% paraformaldehyde (Sigma Aldrich/Merck, Darmstadt, Germany), podocytes were then stained with crystal violet (POCh, Gliwice, Poland), and images of the wounded area were taken on an inverted microscope and were analyzed using Image J with Wound Healing Tool plugin, version 1.53r. (NIH, Bethesda, MD, USA) [31]. Control cells representing initial wound size (0 h) were fixed directly after scratching. The percentage of cell migration area was calculated as area 24 h (or 0 h)/total area of each image. The experiments were performed in triplicate.

### 2.4. Immunofluorescence Staining and Confocal Microscopy

Immunofluorescence studies were performed as described previously [32]. Briefly, podocytes seeded on round glass coverslips (Bionovo, Legnica, Poland) were cultured in NG and HG media as indicated. Following exposure to various treatments, the cells were fixed with 4% paraformaldehyde for 8 min at room temperature, permeabilized (0.3% Triton X-100 in PBS, Thermo Fisher Scientific, Waltham, MA, USA) for 3 min and blocked with blocking solution (2% fetal bovine serum albumin, 0.2% fish gelatine, PBS, Sigma-Aldrich/Merck, Darmstadt, Germany) for 45 min. The permeabilization step was omitted to visualize the surface-bound antibodies. The 60 min incubation with primary antibodies (Table S1) was followed by the subsequent 30 min incubation with secondary antibodies (Table S2). All antibodies were diluted in the blocking solution. Non-specific staining was controlled by replacing the primary antibody with the blocking solution alone. The coverslips were mounted on microscope slides using Fluoroshield TM with DAPI (4′,6-diamidino-2-phenylindole, Sigma-Aldrich/Merck, Darmstadt, Germany). Images were captured with The Opera Phenix^®^ Plus High-Content Screening System (Perkin Elmer, Waltham, MA, USA) and analyzed with Harmony High-Content Imaging and Analysis Software 4.8 (Perkin Elmer, Waltham, MA, USA). The images were merged using the ImageJ software (Version 1.53r, National Institutes of Health, University of Wisconsin, Madison, WI, USA). Scoring for immunofluorescence analysis is presented in Table S3.

### 2.5. RNA Isolation and Reverse Transcription–Quantitative Polymerase Chain Reaction (RT-qPCR)

Total RNA from treated podocytes was extracted and purified with PureLinkTM RNA Mini Kit according to the manufacturer’s instructions (Invitrogen, Carlsbad, CA, USA). The purity and integrity of the extracted RNA were checked with Cytation 3 multimode microplate reader (BioTek, Santa Clara, CA, USA) and analyzed by Gen5 Software (Version 2.04). Quantitative PCR was performed using TaqMan RNA-to-CTTM 1-step KIT (Applied Biosystems, Thermo Fisher Scientific, Waltham, MA, USA), according to the manufacturer’s protocol. Briefly, the RT-PCR reaction mix (TaqMan RT-PCR Mix, TaqMan RT Enzyme Mix, water) was combined with TaqManTM Gene Expression Assay for Fn1 gene-encoding fibronectin, (Assay ID: Mm01256744_m1), TbRI gene-encoding TGF-β receptor1 (Assay ID: Mm00436964_m1), TbRII gene-encoding TGF-β receptor2 (Assay ID: Mm03024091_m1), Actb gene-encoding β-actin (Assay ID: Mm04394036_g1). Then, 50 ng total RNA from each experimental group was added to 7.5 µL of Master Mix (10 µL total volume). PCR reactions were carried out using QuantStudio 3 Real-Time PCR System (Applied Biosystems, Thermo Fisher Scientific, Waltham, MA, USA) and involved the following steps: (1) reverse transcription at 48 °C for 20 min; (2) polymerase activation at 95 °C for 10 min; (3) 40 cycles denaturation (15 s at 95 °C) followed by annealing/extending at 60 °C for 1 min). Relative levels of target gene mRNA expression were normalized to β-actin, and the relative level of mRNA was calculated with the ΔΔ comparative threshold (Ct) method.

### 2.6. Protein Extraction and Western Blot Analysis

The procedure was carried out as previously described [27]. In brief, podocytes were lysed using Pierce TM RIPA Buffer (Thermo Fisher Scientific, Rockford, IL, USA), containing HaltTM Protease & Phosphatase Single-Use Inhibitor Cocktail (Thermo Fisher Scientific, Rockford, IL, USA) and proteins were extracted from the cells according to the manufacturer’s protocol. Total protein concentration was determined by DC Protein Assay (Bio-Rad Laboratories, Hercules, CA, USA). Proteins (30 µg) were separated by CriterionTM TGX Stain-FreeTM Precast Gel electrophoresis and transferred to PVDF membrane (Trans-Blot Turbo, Midi Format, 0.2 µm PVDF) using Trans-Blot Turbo Transfer system (Bio-Rad Laboratories, Hercules, CA, USA). β-actin expression was analyzed to ensure equal protein loading. The membranes were blocked with 5% BSA in TBST buffer (Sigma-Aldrich/Merck, Darmstadt, Germany) for 30 min and incubated overnight with primary antibodies (Table S1). The membranes were washed in TBST and incubated with horseradish peroxidase (HRP)-linked secondary antibody (Table S2). The proteins were then visualized by VisiGloTM Select HRP Substrate Kit (VWR Chemicals, Solon, OH, USA) and imaged using a ChemiDoc MP (Bio-Rad Laboratories, Hercules, CA, USA). Densitometry was performed using ImageLab v2.0 analysis software (Bio-Rad Laboratories, Hercules, CA, USA).

### 2.7. Flow Cytometry Analysis

Podocytes cultured in 6-well plates (90,000 cells/well) were rinsed with PBS, detached by Accutase (Sigma-Aldrich/Merck, Darmstadt, Germany) and centrifuged for 7 min at 400× *g*. Subsequently, the cells were fixed with 4% paraformaldehyde at room temperature for 8 min and blocked with blocking solution (2% FBS, 2% bovine serum albumin, 0.2% fish gelatin, in PBS) for 60 min at room temperature. Finally, the cells were resuspended in cold FACS buffer (2% FBS in PBS) and aliquots of 3 × 10^3^ cells/tube were incubated with a phycoerythrin-conjugated antibody directed against extracellular nephrin epitopes (sc-376522 PE, Santa Cruz, Dallas, TX, USA) for 30 min at 4 °C. To detect total nephrin content, the podocytes were permeabilized with 0.3% Triton X-100 in PBS prior to incubation with primary and secondary antibodies (Tables S1 and S2). To omit debris and cell clumps, gating was performed. Cell fluorescence was analyzed using BD FACSVerse™ Flow Cytometer (BD Biosciences, San Jose, CA, USA) and FlowJo^TM^ Software v10.8.0 (BD Bioscience, San Jose, CA, USA). Background fluorescence, assessed with IgG isotype control, was subtracted from the corresponding samples during analysis.

### 2.8. Statistical Analyses

Statistical tests were performed by using SigmaPlot 11.0 (Systat Software Inc., San Jose, CA, USA) or Statistica 13.3 (TIBCO Software Inc., Santa Clara, CA, USA). All data are shown as means ± SEM and were compared by two-way ANOVA, Mann–Whitney’s *U* test for nonparametric data, or Student’s *t*-tests for paired parametric data.

## 3. Results

### 3.1. Urolithin A Inhibits the TGF-β1-Induced Downregulation of Nephrin

Expression of nephrin, the principal transmembrane component of SD, is suppressed in the hyperglycemic milieu [33]. It has been well documented that, in diabetes, observed overactivity of the TGF-β–dependent system contributes to reducing nephrin expression, which has also been confirmed in the in vitro experiments [34,35,36]. We have shown recently that in podocytes exposed to HG, nephrin expression was restored upon treatment with UA. We also demonstrated that UA modulates endosomal trafficking of nephrin, which could contribute to the mechanisms involved in restoring nephrin by UA [27]. In this study, we investigated whether UA could also modulate the TGF-β1-mediated effects on nephrin expression.

**Figure 2:**
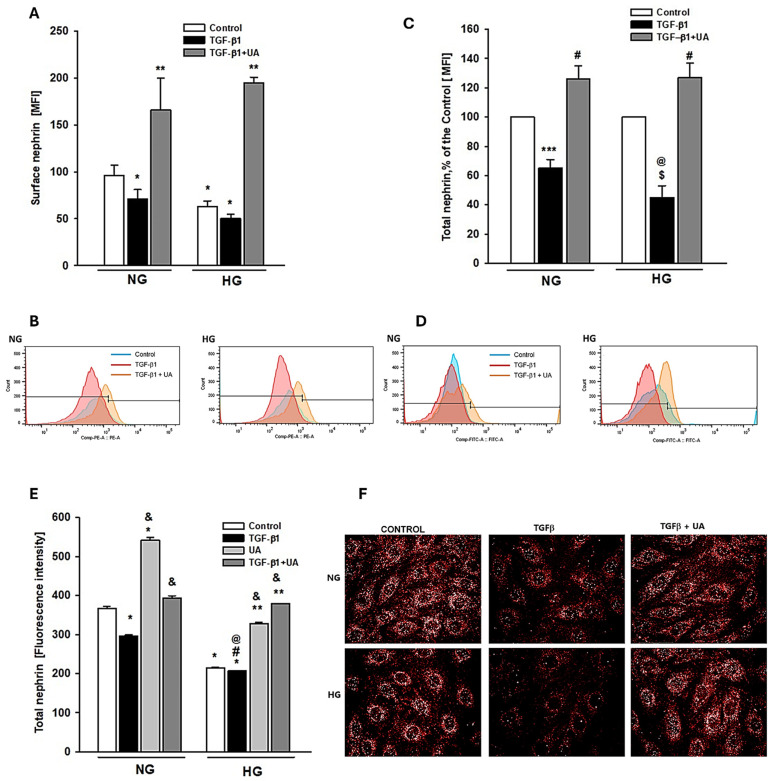
The effect of UA on the TGF-β1 and HG-induced downregulation of nephrin: (**A**,**C**) Quantitative flow cytometry analysis of UA effect on nephrin expression at the podocyte surface (**A**) and total nephrin (**C**). Podocytes cultured for 7 d in normal (5.5 mM, NG) or high (25 mM, HG) glucose were incubated for 24 h with 5 ng/mL TGF-β1 and 10 µM UA, stained with phycoerythrin-conjugated antibody against the extracellular nephrin domain (**A**) or total nephrin (**C**) and analyzed by flow cytometry. (**B**,**D**) Representative histograms showing the effect of UA on surface (**B**) and total (**D**) nephrin expression. (**E**) Quantitative confocal microscopy analysis of total nephrin expression. (**F**) Representative confocal microscopy images of immunofluorescent staining against nephrin. Results show mean ± SEM. Student’s *t*-test and ANOVA test were used to calculate *p*-values. For (**A**,**C**) * *p* < 0.05 vs. NG Control, ** *p* < 0.05 vs. respective TGF-β1 and Control, *** *p* < 0.001 vs. NG Control, # *p* < 0.01 vs. respective TGF-β1, and $ *p* < 0.05 vs. HG Control, @ *p* < 0.05 vs. NG TGF-β1 (*n* = 3–5). For (**E**) * *p* < 0.001 vs. NG Control, ** *p* < 0.001 vs. HG Control, and & *p* < 0.001 vs. respective TGF-β1, # *p* < 0.05 vs. HG Control. 553 cells were analyzed in two independent experiments. MFI: mean fluorescence intensity.

Results of flow cytometry analysis revealed that in HG conditions, incubation of podocytes with TGF-β1 decreased the surface expression of nephrin, which was reversed by co-incubation with UA (Figure 2A). Also, in the NG group, the addition of UA to the TGF-β1-treated cells significantly elevated the surface nephrin, while TGF-β1 alone had no effect. This observation suggests that UA per se could increase the membrane-bound nephrin, which is consistent with our previous findings [27]. On the other hand, total nephrin expression was reduced by TGF-β1 not only in HG, but also in NG cells (Figure 2C–F), while UA reversed the effect.

### 3.2. Urolithin A Inhibits Induced by High Glucose and TGF-β1 Migration of Podocytes

**Figure 3:**
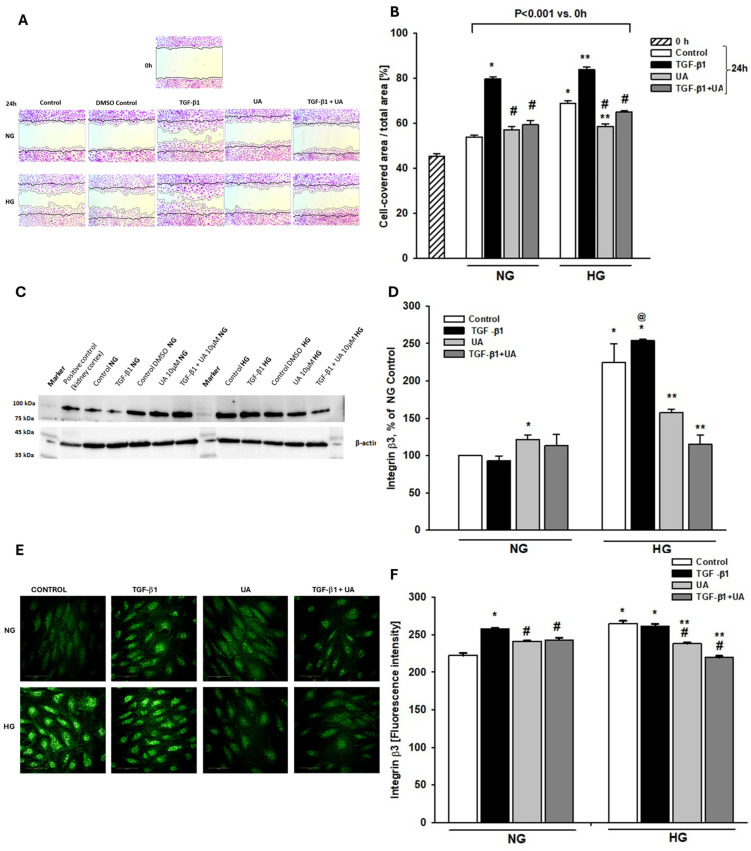
Effects of UA on migratory capacity and expression of β3 integrin in podocytes exposed to TGF-β1 and HG. Podocytes cultured for 7 d in normal (5.5 mM, NG) or high (25 mM, HG) glucose were incubated for 24 h with 5 ng/mL TGF-β1 and/or 10 µM UA: (**A**) Representative image of the wound healing test. After making a scratch in the cell monolayer (time 0), the podocytes were incubated in indicated media for 24 h. (**B**) Quantification of wound healing assay (*n* = 4). (**C**) Representative immunoblot for integrin β3 expression; 30 µg protein samples from total cell lysates were subjected to Western blot analysis followed by quantitative densitometric analysis. (**D**) Quantification of Western blot analyses of β3 integrin expression (*n* = 3). (**E**) Representative confocal microscopy images of immunofluorescent staining against β3 integrin. (**F**) Quantitative confocal microscopy analysis of β3 integrin expression; 754 cells were analyzed in two independent experiments. Results show mean ± SEM. Student’s *t*-test, Mann–Whitney *U* test, and ANOVA were used to calculate *p*-values. * *p* < 0.001 vs. NG Control, ** *p* < 0.001 vs. HG Control, # *p* < 0.001 vs. respective TGF-β1, @ *p* < 0.001 vs. NG TGF-β1.

Nephrin is involved not only in the maintenance of SD architecture but also in the regulation of foot process structure and focal adhesion (FA) dynamics [9]. The rate of FA turnover in turn is a determinant of podocyte motility and contact with the glomerular basement membrane. On the other hand, both TGF-β and high glucose are known to induce podocyte migration [37,38]. We presumed, therefore, that modulation by UA of TGF-β-dependent and -independent nephrin expression could influence the ability of podocytes to migrate. As shown in Figure 3A,B, the wound healing tests revealed that high glucose alone triggered podocyte migration, while TGF-β1 potentiated motility of podocytes exposed to both NG and HG conditions. UA apparently reduced the ability to migrate of high glucose-stimulated podocytes, as well as podocytes treated with TGF-β1. However, no effect of UA alone was observed in the NG group.

### 3.3. Integrin β3 Expression Is Modulated by Urolithin A

Based on the above-mentioned results, it seemed likely that the suppression of the migratory capacity of podocytes by UA was mediated by affecting a common factor triggered by high glucose, as well as by TGF-β1. It has been well documented that increased podocyte motility occurs following integrin β3 activation [39,40,41]. Moreover, integrin β3 in podocytes is upregulated by both, the hyperglycemic milieu [42,43] and by TGF-β [38]. Thus, our next aim was to check if the observed-by-us UA-mediated-reduced migratory capability of podocytes was due to the modulation of integrin β3 expression. The results of Western blot (Figure 3C,D) and quantitative confocal image analyses (Figure 3E,F) consistently demonstrate that induction by TGF-β1 and by a high-glucose increase in migration was paralleled by respective upregulation of integrin β3 expression. Likewise, inhibition by UA of podocyte motility was accompanied by the downregulation of integrin β3 protein.

### 3.4. Urolithin A Modulates Fibronectin Expression

**Figure 4:**
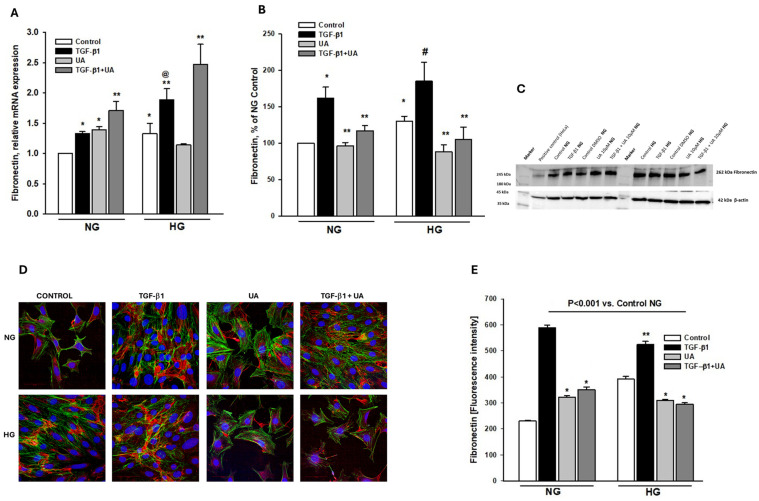
Modulation by UA of fibronectin expression in podocytes exposed to TGF-β1 and HG. Podocytes cultured for 7 d in normal (5.5 mM, NG) or high (25 mM, HG) glucose were incubated for 24 h with 5 ng/mL TGF-β1 and/or 10 µM UA: (**A**) Results of quantitative RT-PCR analysis for fibronectin. Relative levels of mRNA were normalized to β-actin (*n* = 4). (**B**) Quantification of Western blot analyses of fibronectin expression (*n* = 3–4). (**C**) Representative immunoblot for fibronectin expression; 30-µg protein samples from total cell lysates were subjected to Western blot analysis followed by quantitative densitometric analysis. (**D**) Representative confocal microscopy images of immunofluorescent staining showing fibronectin (red), F-actin (green), and counterstained with DAPI (blue). (**E**) Quantitative confocal microscopy analysis of fibronectin expression; 758 cells were analyzed in two independent experiments. Results show mean ± SEM. Student’s *t*-test, Mann–Whitney *U* test, and ANOVA test were used to calculate *p*-values. For (**A**) * *p* < 0.001 vs. NG Control, ** *p* < 0.01 vs. respective Control, @ *p* < 0.01 vs. NG TGF-β1. For (**B**) * *p* < 0.01 vs. NG Control, ** *p* < 0.02 vs. respective TGF-β1, # *p* < 0.02 vs. HG Control. For (**E**) * *p* < 0.001 vs. respective Control and TGF-β1, ** *p* < 0.001 vs. HG Control.

The loss of expression of typical epithelial markers such as nephrin, along with increased podocyte migratory potential, is typical for the epithelial-to-mesenchymal transition (EMT) that occurs under conditions of hyperglycemia and upon TGF-β activation [44,45,46]. Additionally, a switch in the cell phenotype is also characterized by the upregulation of mesenchymal state markers, such as fibronectin [45,47]. Thus, to assess whether the observed-by-us changes in podocytes were associated with EMT, we examined the expression of fibronectin and investigated whether it was modulated in the presence of UA. High glucose significantly elevated fibronectin mRNA (Figure 4A), which was paralleled by respective increases in protein levels (Figure 4B,E). Upon treatment with TGF-β1, fibronectin mRNA, as well as protein levels, was increased in both the NG and HG groups. Interestingly, in the NG conditions, exposure of podocytes to UA increased fibronectin mRNA expression (Figure 4A). Yet, the high glucose-induced elevation of fibronectin mRNA and protein expression was significantly reduced by UA, reaching the level of the NG control. Upon the addition of UA to the TGF-β1-treated cells, the elevated-by-TGF-β1 level of fibronectin protein apparently decreased (Figure 4B,E), whereas the simultaneous prominent increase in respective mRNA expression was observed (Figure 4A).

### 3.5. Urolithin A Affects the Expression of TGF-β Receptors

**Figure 5:**
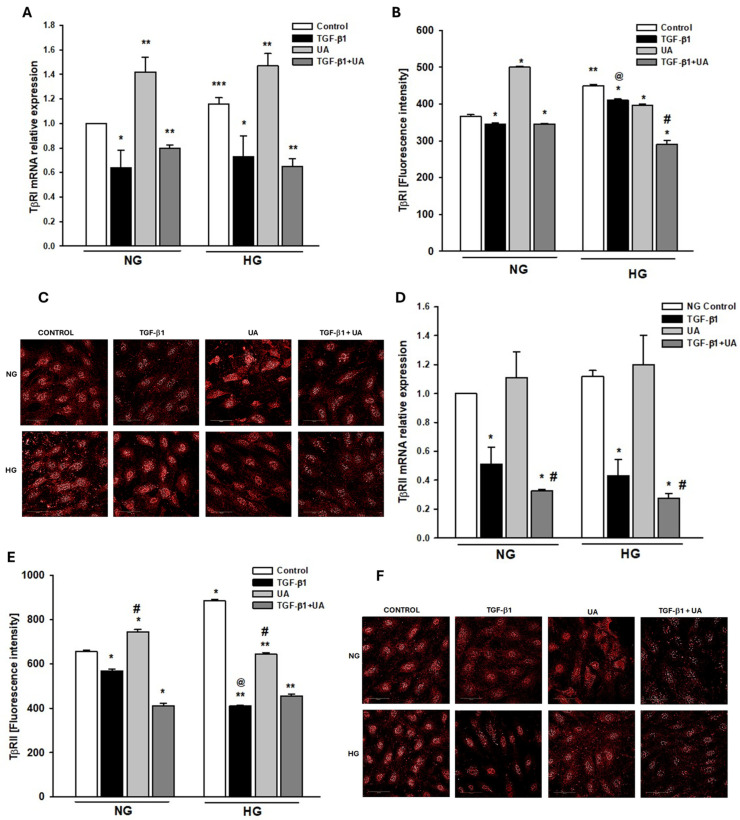
Effects of UA on the expression of TGF-β1 receptors TβRI and TβRII. Podocytes cultured for 7 d in normal (5.5 mM, NG) or high (25 mM, HG) glucose were incubated for 24 h with 5 ng/mL TGF-β1 and/or 10 µM UA: (**A**) Results of quantitative RT-PCR analysis for TβRI. Relative levels of mRNA were normalized to β-actin (*n* = 3). (**B**) Quantitative confocal microscopy analysis of TβRI expression; 810 cells were analyzed in two independent experiments. (**C**) Representative confocal microscopy images of immunofluorescent staining against TβRI. (**D**) Results of quantitative RT-PCR analysis for f TβRII. Relative levels of mRNA were normalized to β-actin (*n* = 3). (**E**) Quantitative confocal microscopy analysis of TβRII expression; 840 cells were analyzed in two independent experiments. (**F**) Representative confocal microscopy images of immunofluorescent staining against TβRII. Results show mean ± SEM. Student’s *t*-test and ANOVA test were used to calculate *p* values. For (**A**) * *p* < 0.05 vs. respective Control, ** *p* < 0.001 vs. respective Control, *** *p* < 0.005 vs. NG Control. For (**B**) * *p* < 0.001 vs. respective Control, ** *p* < 0.001 vs. NG Control, # *p* < 0.001 vs. TGF-β1, @ *p* < 0.001 vs. NG TGF-β1. For (**D**) * *p* < 0.05 vs. respective Control, # *p* < 0.01 vs. UA. For (**E**) * *p* < 0.001 vs. NG Control, ** *p* < 0.001 vs. HG Control, # *p* < 0.001 vs. respective TGF-β1 and UA, @ *p* < 0.001 vs. NG TGF-β1.

The TGF-β signaling pathway is initiated by the sequential binding of TGF-β to its type II (TβRII) and type I (TβRI) receptors on the cell membrane. To find out the mechanisms by which UA modulated the TGF-β1-dependent effects, we next checked whether the expression of TGF-β receptors TβRI and TβRII was modulated in our experimental conditions and whether it was affected by urolithin A. Results showed that high glucose upregulated TβRI and TβRII protein expression (Figure 5B,E), with a significant increase in TβRI mRNA (Figure 5A,D). In the NG, as well as in the HG conditions, treatment of podocytes with TGF-β1 resulted in marked downregulation of mRNA for both receptors, which, except for TβRII in NG, was accompanied by reduced protein level, as compared to the respective control. To our surprise, UA elevated the TβRI and TβRII mRNA in both NG and HG groups. Yet, respective proteins’ expressions were downregulated, except for TβRI in the NG cells. Despite the apparent stimulatory effect of UA on TβR mRNA expression, the TGF-β1-induced reduction in the TβRI mRNA level was unaffected upon the addition of UA, whereas, in the case of TβRII, the decline was even potentiated. This was reflected by respective changes in protein expression, both in NG and in HG groups.

### 3.6. Urolithin A Reduces the TGF-β1-Dependent Smad2 Activation

**Figure 6:**
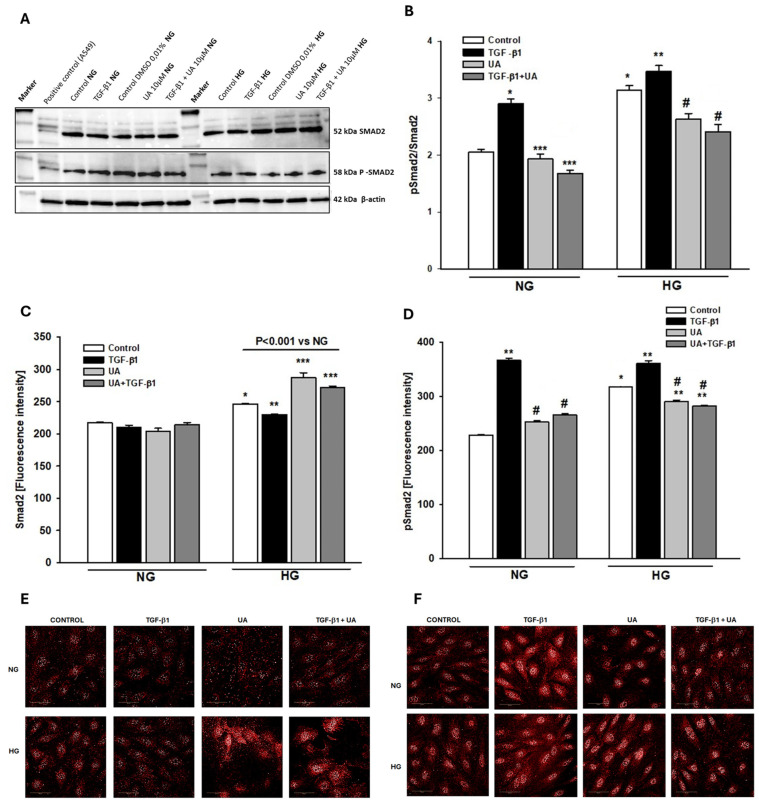
Effects of UA on Smad2-dependent signaling of TGF-β1. Podocytes cultured for 7 d in normal (5.5 mM, NG) or high (25 mM, HG) glucose were incubated for 24 h with 5 ng/mL TGF-β1 and/or 10 µM UA: (**A**) Representative immunoblots for expression of Smad2 and phospho-Smad2 (pSmad2); 30-µg protein samples from total cell lysates were subjected to Western blot analysis followed by quantitative densitometric analysis. (**B**) Quantification of Western blot analyses of pSmad2/Smad2 expression ratio (*n* = 3). (**C**) Quantitative confocal microscopy analysis of Smad2 expression; 550 cells were analyzed in two independent experiments. (**D**) Quantitative confocal microscopy analysis of pSmad2 expression; 550 cells were analyzed in two independent experiments. (**E**) Representative confocal microscopy images of immunofluorescent staining against Smad2. (**F**) Representative confocal microscopy images of immunofluorescent staining against pSmad2. Results show mean ± SEM. Student’s *t*-test and ANOVA test were used to calculate *p*-values. For (**B**) * *p* < 0.001 vs. NG Control, ** *p* < 0.03 vs. HG Control, *** *p* < 0.01 vs. NG TGF-b1, # *p* < 0.03 vs. HG TGF-b1. For (**C**) * *p* < 0.001 vs. NG Control, ** *p* < 0.01 vs. HG Control, *** *p* < 0.001 vs. Control and TGF-b1. For (**D**) * *p* < 0.001 vs. NG Control, ** *p* < 0.001 vs. respective Control, # *p* < 0.01 vs. respective TGF-b1.

The binding of the TGF-β ligand to its receptors activates downstream signal transduction, which is predominantly mediated by the Smad family of proteins. Consequently, cytoplasmic Smad2 and Smad3 are phosphorylated, which is the crucial intermediate step to inducing the biological response [48]. Since phosphorylation by TGF-β1 of Smad2 in podocytes has been demonstrated to be involved in various intracellular changes [19,49,50], we examined whether UA could modulate the TGF-β1-dependent effects by affecting Smad2 signaling. In both, NG and HG conditions, treatment of podocytes with TGF-β1 increased the level of Smad2 phosphorylation (Figure 6B,D). Additionally, high glucose alone elevated not only pSmad2 but also Smad 2 expression (Figure 6C,D). In both cases, upon the addition of UA, we observed a significant drop in the expression of phosphorylated Smad2, which indicates that UA inhibited activation of the signal transducer.

## 4. Discussion

Presented in this study results demonstrate that urolithin A counteracts the phenotypic changes induced in podocytes by TGF-β1 and high glucose. We show that under conditions mimicking the diabetic milieu, UA suppresses podocyte motility, inhibits the Smad2-dependent TGF-β1 signaling, and opposes the epithelial-to-mesenchymal transition.

Podocyte injury and loss lead to irreversible changes in the glomerular filtration barrier (GFB), making these cells crucial in the progression of diabetic kidney disease (DKD) [51,52]. Typically, podocyte impairment is manifested by effacement of foot processes, which is associated with loss of SD components, dedifferentiation, EMT, and finally, detachment and loss of viable, apoptotic, or necrotic cells [53,54,55,56].

EMT is a complex process mediating podocyte dysfunction in diabetes as well as in non-diabetic chronic kidney diseases such as renal fibrosis or focal segmental glomerulosclerosis (FSGS) [57,58]. During EMT, epithelial features of podocytes, including nephrin expression, are lost and the cells acquire mesenchymal features that are manifested by increased migratory properties and expression of proteins such as fibronectin, α-smooth muscle actin, and others [59,60]. The in vivo and in vitro studies show that among different microenvironmental stimuli, TGF-β is a potent inducer of EMT in podocytes under normal [20,45,61], as well as under high glucose conditions [62,63]. High glucose concentration induces EMT in podocytes not only through the activation of TGF-β signaling but also through several other molecular mechanisms [44,64,65]. EMT is considered to be the major pathomechanism underlying podocytopenia in diabetic kidney [59].

Our results indicate that TGF-β1 and HG, separately and in concert, induced, in podocytes, changes typical for EMT, which was abolished upon treatment with UA. Both these factors independently increased the ability of podocytes to migrate (Figure 3A,B), which is consistent with previous reports [38,66]. Similarly, the expression of nephrin, the key component of SD and marker protein of podocytes, was separately downregulated by both these factors. However, the effect induced by TGF-β1 was significantly augmented in HG conditions (Figure 2). On the other hand, expression of fibronectin was strongly increased by TGF-β1, as well as by high glucose (Figure 4), and the effect was enhanced when podocytes were exposed to both factors simultaneously (Figure 4A). In the presence of UA, the impact of TGF-β1 was apparently suppressed, while the changes elicited by prolonged exposure of the cells to HG were reversed.

In diabetic kidneys, the hyperglycemic milieu and TGF-β act on podocytes simultaneously [67]. Under HG conditions, increased interaction of podocytes with TGF-β results from overproduction and secretion by glomerular endothelial and mesangial cells of TGF-β and TGF-β mRNA-containing exosomes [68]. Moreover, podocytes can also produce TGF-β acting in an autocrine manner, and this phenomenon occurs also in the normoglycemic milieu [69,70,71]. In our in vitro experiments, most of the features investigated here were modified by TGF-β1 not only in HG, but also in NG conditions, while high glucose concentration was an independent factor affecting the cells. However, UA hindered the activity of TGF-β1 and reversed the changes induced by prolonged preincubation of podocytes in HG.

In physiological conditions, podocytes display a limited ability to migrate, which allows them to withstand injurious stimuli such as inflammatory or mechanical stress [7,72,73]. However, excessive stress leads to dysregulated cell motility, which is tightly associated with disruption of SD, proteinuria, and podocyte detachment [73,74]. We show here that in HG conditions and upon stimulation with TGF-β1, increased podocyte motility (Figure 3B) was associated with the elevation of β3 integrin, whereas abolishment by UA of cell migration was paralleled by downregulation of β3 integrin (Figure 3D,F). These results are consistent with previously published reports revealing that there is an inverse relationship between the expression of β3 integrin and the capability of podocytes to migrate [38].

**Figure 7:**
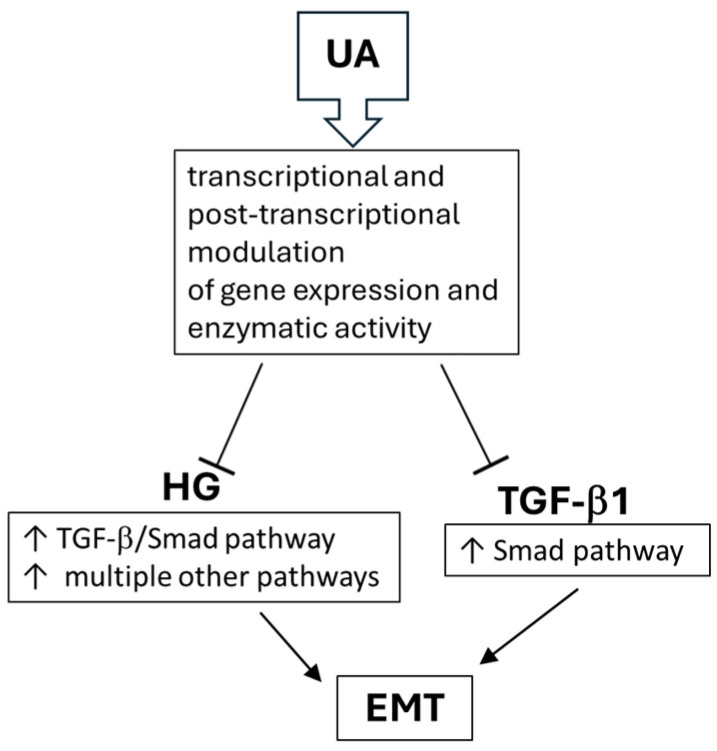
Proposed mechanisms of UA inhibition of HG-dependent and -independent EMT in podocytes.

We demonstrate here that typical for EMT responses of podocytes to TGF-β1, such as the decrease in nephrin and the increase in integrin β3 or fibronectin were enhanced in the hyperglycemic milieu. In both NG and HG conditions, UA significantly opposed the detrimental effects of the cytokine, which was at least partly mediated by interrupting the TGF-β1 signal transduction. As shown in Figure 5, high glucose concentration increased the expression of TβRI and TβRII receptors, which is consistent with previous reports [75,76,77]. However, in the presence of UA, we noted the rise in TβRI mRNA with a concomitant drop in protein expression. In our recent study, we noted a similar discrepancy between mRNA and protein during the quantification of the UA-dependent modulation of nephrin expression [27]. Such inconsistency is a frequent phenomenon [78] and suggests that urolithin A could induce post-transcriptional changes in the expression of proteins. Upon treatment with TGF-β1, both TβRI and TβRII receptors were downregulated. Reports regarding the influence of TGF-β on its own receptors are conflicting and reveal up- as well as downregulation of TβRI and TβRII [79,80]. However, it has also been found that the final effect depends on the duration of exposure of the cells to TGF-β [81]. Brief administration of TGF-β resulted in upregulation of its own receptors, whereas prolonged incubation reduced expression of TβRI and TβRII. In our experiments, the podocytes were exposed to TGF-β1 for 24 h, corresponding to the prolonged incubation, which resulted in a decrease in the expression of receptors. Co-incubation of HG-treated podocytes with TGF-β1 along with UA further reduced the TβRI expression (Figure 5B), which, most likely, contributed to diminished responsiveness of the TGF-β receptor system to stimulation by the ligand. Moreover, we show here that UA also interrupts the TGF-β1 signal transduction downstream to the receptors. The TGF-β/Smad signaling pathway is one of the most important signal pathways mediating EMT and apoptosis in podocytes [65,82]. Formation of the TGF-β/TβRII/TβRI complex triggers the downstream phosphorylation and activation of Smad2/3 proteins, which is crucial for further transduction of the signal to the nucleus [48]. We have proven that UA reduces the mediation by HG and the TGF-β1 increase in the pSmad2/Smad2 ratio (Figure 6B). In the presence of HG and TGF-β1, the expression of not only the phosphorylated form (pSmad2) but also of the unphosphorylated Smad2 was elevated (Figure 6C,D). Yet, in HG conditions, UA stimulated an even bigger increase in Smad2, which was accompanied by strong suppression of pSmad2 expression. The proposed mechanisms of UA inhibition of HG-dependent and -independent EMT in podocytes are presented in Figure 7.

Detailed mechanisms by which UA opposes the effects of high glucose and TGF-β remain to be established. It is known so far that UA is capable of regulating the expression of various proteins by modulating transcription and post-transcriptional processes [28,83,84]. The broad range of beneficial biological activities exerted by urolithins, first of all by UA, has been listed lately in the comprehensive review by Hasheminezhad et al. [26]. Recently, inhibition by UA of EMT in cancer cells [84,85] and inhibition of TGF-β signaling in renal epithelial cells [86] were reported. However, relatively few research results refer to the influence of UA on renal disorders, with only a few publications discussing its impact on podocytes. Our results reveal that in conditions mimicking diabetes, UA inhibits the EMT-associated changes in podocytes. Moreover, we show here that also in the normoglycemic milieu, UA opposes the effects of TGF-β1, the principal mediator in the development of kidney fibrosis, glomerulosclerosis, and CKD [12,87,88].

## 5. Conclusions

In summary, our in vitro experiments demonstrated that in conditions mimicking diabetes, UA protects podocytes from phenotypic changes underlying the development of proteinuria. Moreover, UA also inhibits the negative effects of TGF-β1 in podocytes cultured in the normoglycemic milieu. These findings indicate that UA has the potential to become a candidate drug for treating podocyte-related renal diseases.

## Data Availability

All study data can be found within this article. Data can be made available upon request.

## Supplementary Materials

The following supporting information can be downloaded at: https://www.mdpi.com/article/10.3390/jpm14090914/s1, Table S1: Primary antibodies; Table S2: Secondary antibodies.
