# Supplementary material for: Urolithin A Ameliorates the TGF Beta-Dependent Impairment of Podocytes Exposed to High Glucose
Source: J Pers Med. 2024 Aug 28;14(9):914. doi: 10.3390/jpm14090914 (PMC11433157; doi:10.3390/jpm14090914)
Supplement: Supplementary file 1 [file jpm-14-00914-s001.zip › jpm-3110061-supplementary.pdf]

Table S1. Primary antibodies.

Primary Antibody	Application and Dilution	Catalog number	Source
Nephrin (G-8)	FC 1:200, IF 1:50	sc-376522	Santa Cruz Biotechnology, U.S.
Nephrin	FC 1:100, WB 1:500	ab216341	Abcam, Cambridge, UK
Integrin-β3	IF 1:50 WB 1:1000	sc-46655	Santa Cruz Biotechnology, U.S.
Fibronectin	WB 1:1000	ab2413	Abcam, Cambridge, UK
TβRI	IF 1:100	PA5-3263	Invitrogen, Thermo Fisher Scientific, U.S.
TβRII	IF 1:100	ab61213	Abcam, Cambridge, UK
Smad2	IF 1:100 WB 1:1000	51-1300	Invitrogen, Thermo Fisher Scientific, U.S.
p-Smad2	IF 1:100 WB 1:1000	44-244G	Invitrogen, Thermo Fisher Scientific, U.S.

WB: Western blot, **IF**: Immunofluorescence

Table S2. Secondary antibodies.

Secondary Antibody	Application and Dilution	Catalog number	Source
Goat anti-rabbit/Alexa Fluor 647	IF 1:100	ab150079	Abcam, Cambridge, UK
Goat anti-mouse/Alexa Fluor 488	IF 1:100	ab150113	Abcam, Cambridge, UK
MFP™-DY-490-Phalloidin	IF 1:100	MFP-D490-33	MoBiTec Molecular Biotechnology, Germany
Anti-rabbit IgG, HRP-linked	WB 1: 2000	7074S	Cell Signaling Technology, U.S.
Anti-mouse IgG, HRP-linked	WB 1: 2000	7076	Cell Signaling Technology, U.S.

WB: Western blot, **IF**: Immunofluorescence, **HRP-linked**: Horseradish peroxidase-linked

Table S3. The strength of binding of the monoclonal antibody (in combination with a fluorescently labeled secondary antibody) to each individual protein represented as the Z-score. The Z-score is described in units of standard deviations (SDs) above the mean value of all signals generated on that array.

	Nephrin	β3 Integrin	Fibronectin	TβRI	TβRII	Smad2	pSmad2
**Control, NG**	3.7	20.8	10.4	3.2	9.6	4.3	4.9
**TGF-β1, NG**	5.3	4.6	5.3	6.3	5.4	3.1	5.0
**UA, NG**	-	5.9	6.5	4.4	2.7	2.3	4.7
**TGF-β1 + UA, NG**	3.1	4.0	6.9	5.7	5.6	2.5	4.0
**Control, HG**	11.6	6.7	7.4	4.4	4.8	5.2	5.4
**TGF-β1, HG**	4.7	4.3	5.4	5.0	3.2	4.3	3.7
**UA, HG**	-	8.4	7.7	5.8	4.6	3.6	4.4
**TGF-β1 + UA, HG**	4.3	7.0	8.1	3.5	4.6	4.5	4.2
